# Supplementary material for: Simultaneous assessment of mechanical and electrical function in Langendorff-perfused ex-vivo mouse hearts
Source: Front Cardiovasc Med. 2023 Nov 8;10:1293032. doi: 10.3389/fcvm.2023.1293032 (PMC10663365; doi:10.3389/fcvm.2023.1293032)
Supplement: Supplementary file 1 [file Datasheet1.pdf]

## *Supplementary Material*

### 1 Supplementary Figures and Tables

### 1.1 Supplementary Figures

**Supplementary Figure 1**

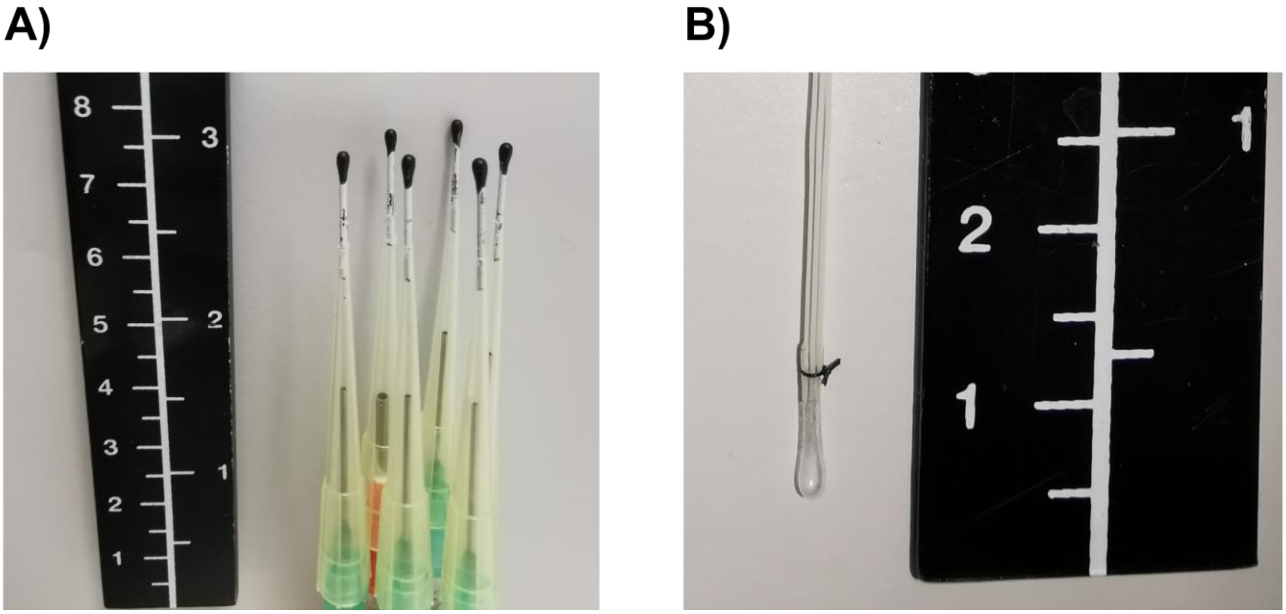


**Supplementary Figure 1. Fabrication of the left ventricular silicone balloon.** Mold of the balloons made of pipette tips and the bristles of a hairbrush. Scale (cm left / inches right). B) Finished silicone balloon connected to a plastic tube and secured with a thin string. Scale (cm left / inches right).

**Supplementary Figure 2**

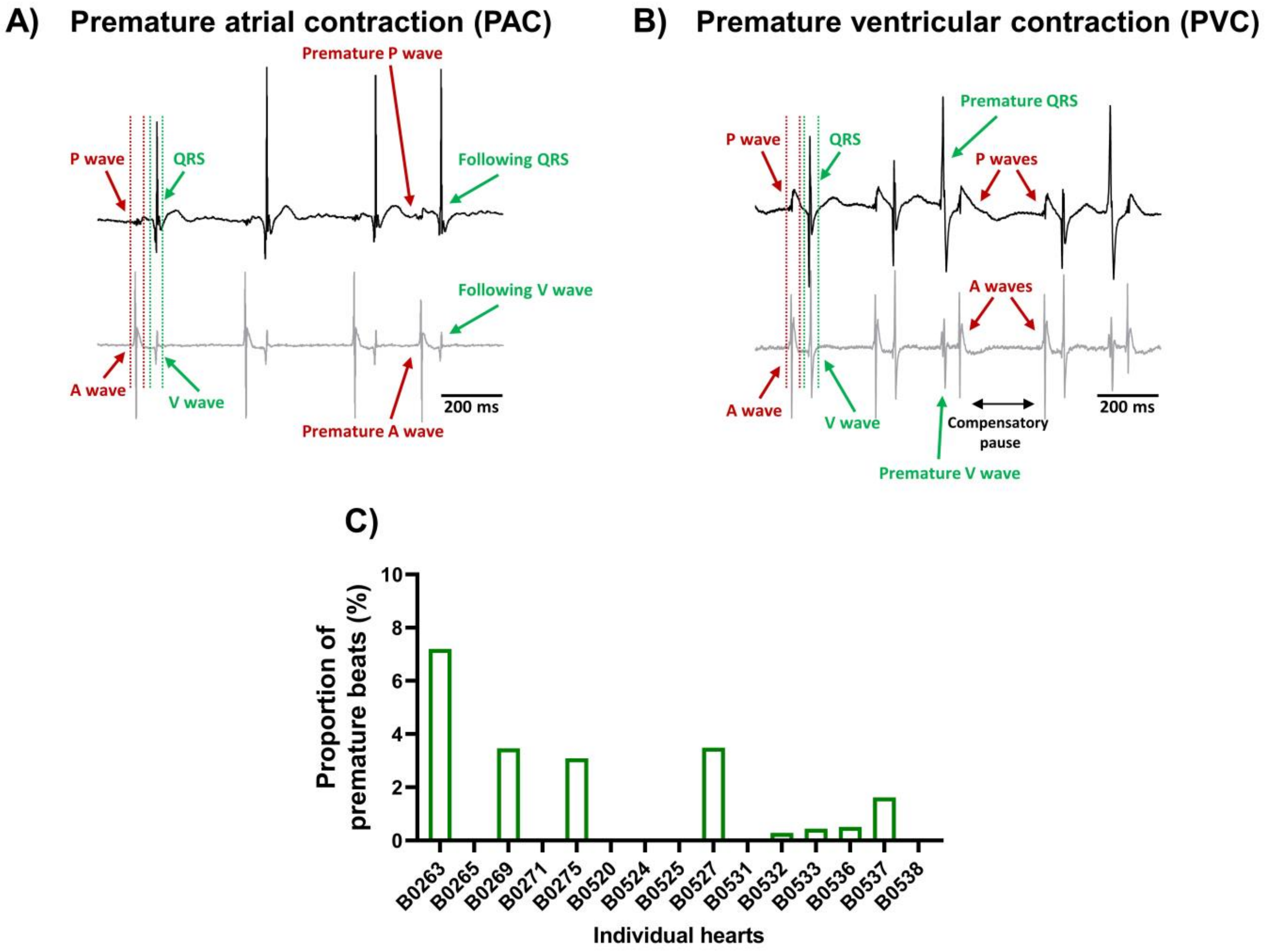


**Supplementary Figure 2. Spontaneous isolated premature beats at baseline.** Example recordings of the electrocardiogram trace (top, black) and one signal trace (channel 6, see Figure 2) from the octapolar catheter (bottom, gray) of a premature atrial contraction (PAC) (A) and a premature ventricular contraction (PVC) (B) at baseline. Red and green arrows indicate atrial and ventricular signals, respectively. (C) Proportion of premature beats normalized to the total number of beats during the considered time-window at baseline.

**Supplementary Fig. 3**

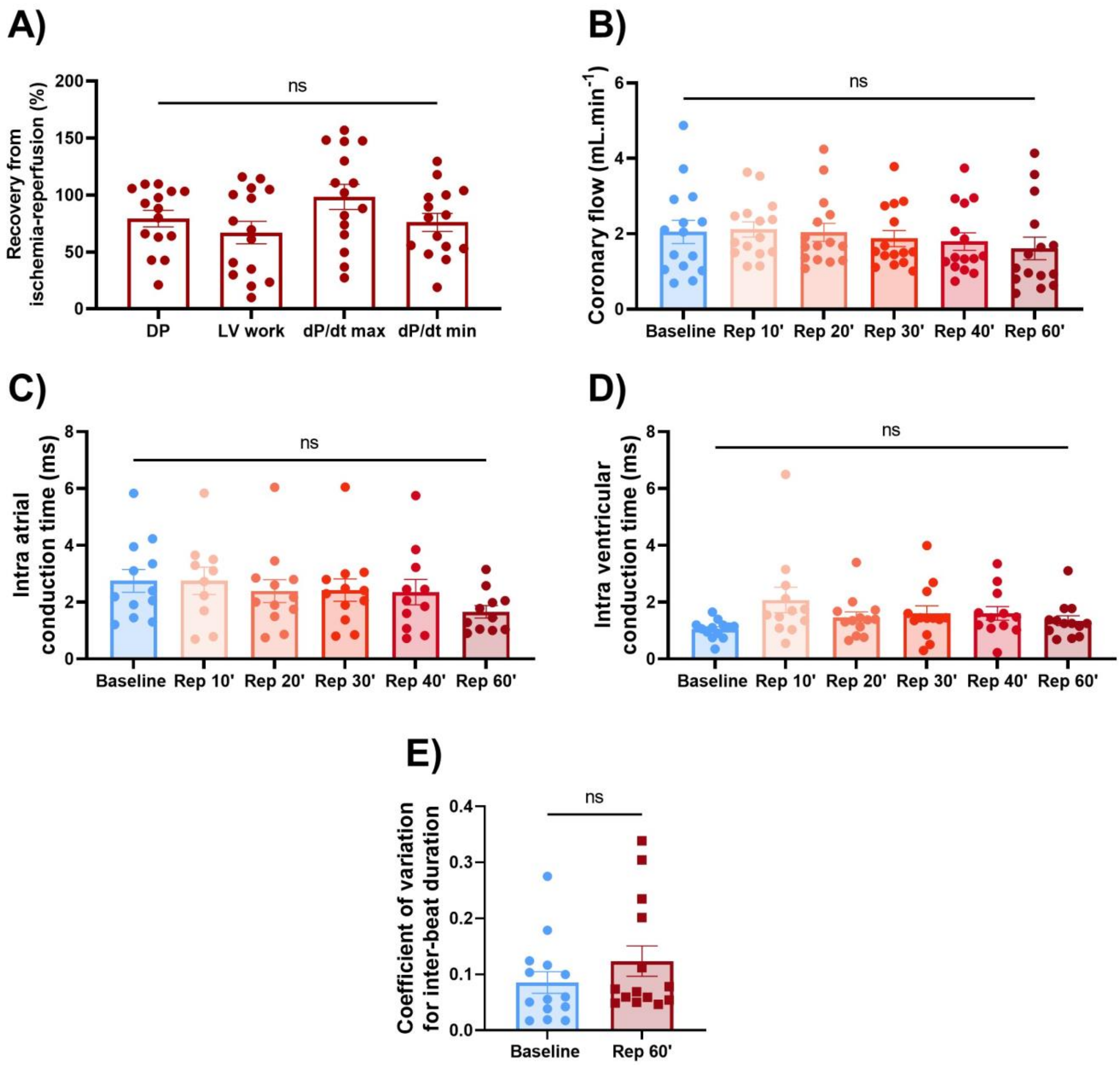


**Supplementary Figure 3. Effects of ischemia-reperfusion on mechanical and electrical function.** (A) Average percentage recovery from ischemia at the end of reperfusion normalized to baseline values for developed pressure, LV work and maximal velocity of contraction (dp/dt max) and relaxation (dP/dt min) (N=15). Average coronary flow (B) and atrial (C) and ventricular (D) conduction times at baseline and 10, 20, 30, 40, and 60 minutes of reperfusion (Rep 10', 20', 30', 40', 60') (N=15) (E) Average coefficient of variation for inter-beat duration between baseline and 60 minutes of reperfusion (Rep 60'). *ns = non-significant with one-way ANOVA followed by Dunnett's multiple comparison test (A-D) or non-parametric paired t-test (Wilcoxon test) (E).*

**Supplementary Fig. 4**

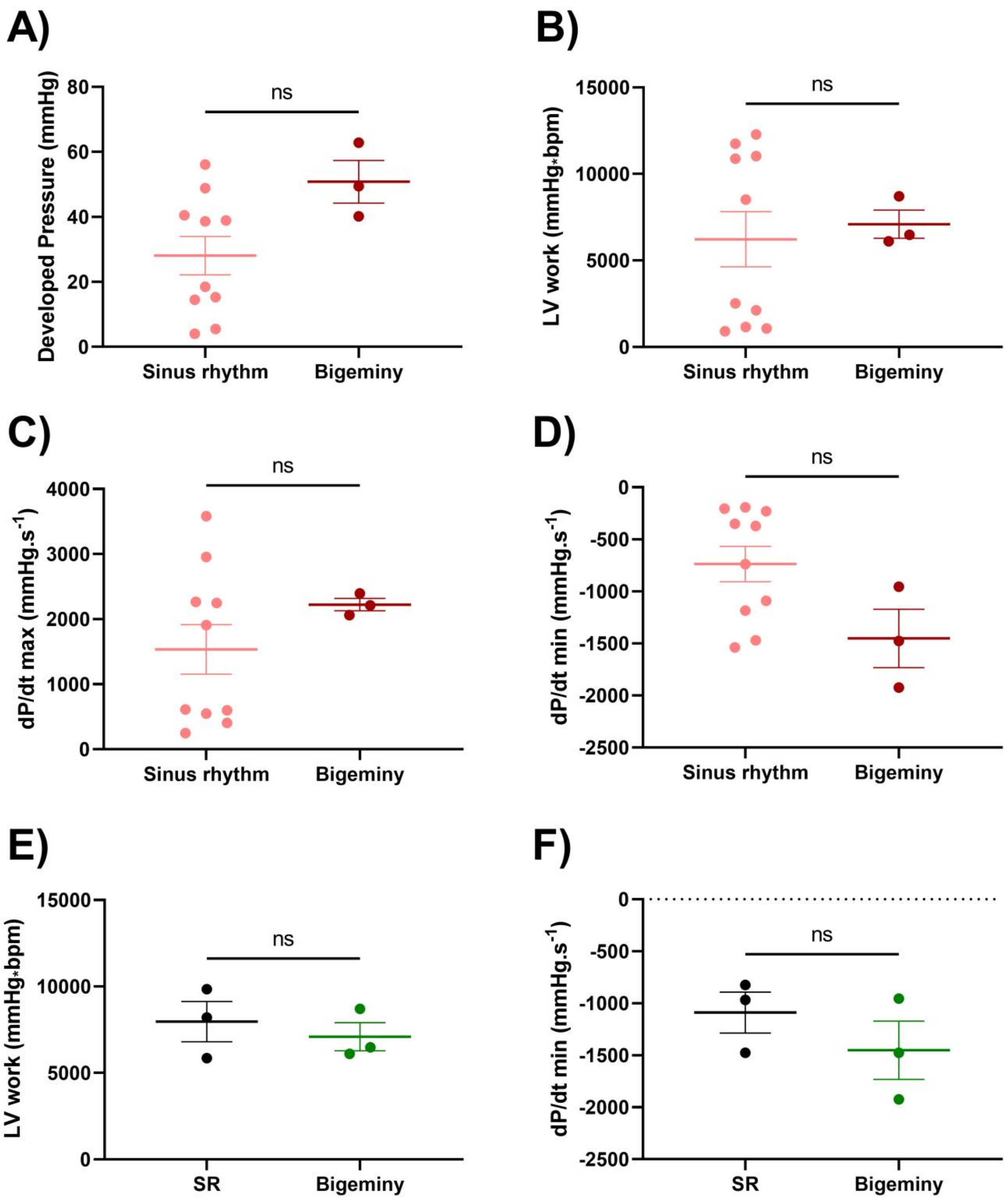

**Supplementary Figure 4. Effects of arrhythmia on mechanical function after global ischemia-reperfusion.** Average developed pressure (A), left ventricular (LV) work (B), and maximal velocity of contraction (dP/dt max) (C) and relaxation (dP/dt min) (D) between individual hearts presenting with sinus rhythm or bigeminy at 60 minutes reperfusion (N=13). Average LV work (E) and maximal velocity of relaxation (F) between episodes of sinus rhythm (black) or bigeminy (green) in the same hearts (N=3). *ns = non-significant with unpaired (A-D) or paired (E-F) t-test.*

frontiers
